# Injection, confinement, and diagnosis of electrons and positrons in a permanent magnet dipole trap

**DOI:** 10.1140/epjd/s10053-024-00821-x

**Published:** 2024-12-05

**Authors:** J. von der Linden, S. Nißl, A. Deller, M. Singer, N. Belmore, C. P. Hugenschmidt, T. Sunn Pedersen, H. Saitoh, E. V. Stenson

**Affiliations:** 1 Max Planck Institute for Plasma Physics, 85748 Garching & 17491 Greifswald, Germany; 2 Technische Universität München, 85748 Garching, Germany; 3 University of California San Diego, La Jolla, CA 92093 USA; 4 Applied Materials, 85551 Heimstetten, Germany; 5 TicketSwap, 1012 KL Amsterdam, The Netherlands; 6 Type One Energy Group, Madison, WI 53703 USA; 7 University of Tokyo, Kashiwa, Chiba 277-8561 Japan

## Abstract

**Abstract:**

Prerequisites for the goal of studying long-lived, magnetically confined, electron–positron pair plasmas in the laboratory include the injection of both species into the trap, long trapping times, and suitable diagnostic methods. Here we report recent progress on these tasks achieved in a simple dipole trap based on a supported permanent magnet. For the injection of electrons, both an $\textbf{E}\times B$ drift technique (of a $\sim$2–$\upmu$A, 6-eV beam) and “edge injection” (from a filament emitting a few mA and biased to some tens of volts) have been demonstrated; the former is suitable for low-density beams with smaller spatial and velocity spreads, while the latter employs fluctuations arising from collective behavior. To diagnose the edge-injected electrons, image potentials and currents induced on a wall probe, the magnet case, and wall electrodes were measured. Confinement of drift-injected positrons, measured experimentally, exhibited at least two well-separated timescales. Simulations reproduced this qualitatively, using a simple model of elastic collisions with residual background gas, and point to small adjustments for increasing trapping times. In a major upgrade to diagnostic capabilities, 25 bismuth germanate detectors, placed in three reentrant ports, are able to localize annihilation gammas, which will be used in future experiments to distinguish between different loss channels.

**Graphical abstract:**

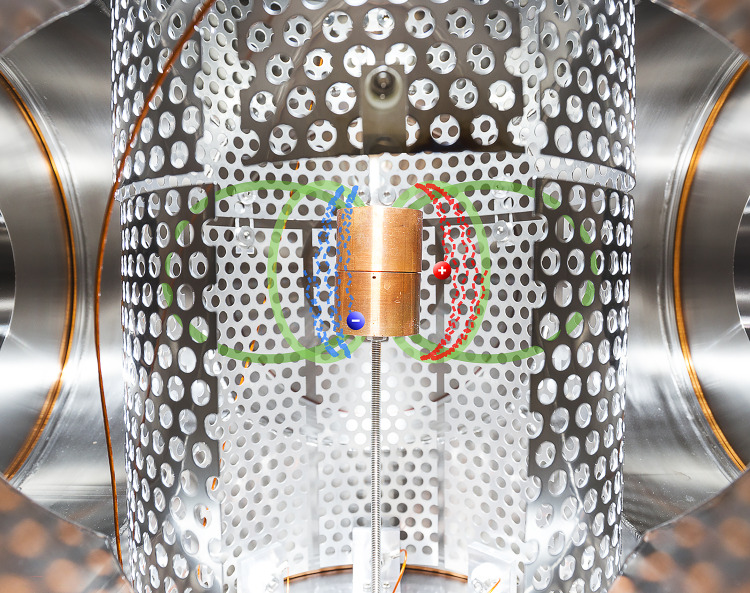

## Introduction

A “pair plasma” is an idealized type of plasma, comprising equal amounts of positively and negatively charged particles with identical mass and temperature. (Both species must also be at sufficiently high densities and low temperatures such that they exhibit collective effects that compete with externally applied electric and/or magnetic fields—i.e., the definition of a plasma.) This perfect symmetry drastically changes the response of the pair plasma to some types of perturbations; the number of time and length scales involved is reduced [1], and electrostatic instabilities may be suppressed [2, 3]. Over the last four decades [4], hundreds of theoretical and numerical investigations of pair plasmas have been conducted (see, e.g., partial lists in [1, 5]); however, experimental investigations are still in their nascence. Laser-driven relativistic unmagnetized positron–electron beams have achieved charge neutrality [6] and small skin depths [7], and in the weakly magnetized regime heavier carbon fullerene pair plasma with small Debye lengths has been produced [8, 9]. The collective behavior of magnetized electron–positron pair plasma has yet to be observed in the laboratory [10, 11].

To trap an electron–positron plasma, both species must be injected into a magnetic field geometry with good confinement properties; good confinement, however, tends to inhibit or exclude efficient injection. The property that collisionless charged particles do not, on average, drift radially inward or outward from confining magnetic surfaces is the definition of an omnigenous magnetic field [12]; in systems with a continuous symmetry (i.e., an ignorable coordinate), this occurs as a direct consequence of the conservation of canonical momentum. There are nevertheless ways to populate such a field from either an internal or external charge source.

In fusion experiments, internal sources are commonly used, such as breaking down gas, ablating pellets, or ionizing neutral beams. Adapting internal generation techniques to pair plasma involves generating the pairs from laser or electron interactions with a target placed inside the magnetic geometry [11] or by ionizing positronium (the bound state of an electron and a positron) [10, 13]. The latter approach is technically challenging but would offer the advantage of guaranteeing charge neutrality and minimizing perturbations to those particles that are already in the trap.

This work focuses on techniques for injecting externally generated charges into a confinement field, including

1. *drift injection*: applying electric fields perpendicular to the confining magnetic fields, causing charged particles to $\textbf{E}\times B$ drift into the trap [14–18], and
2. *edge injection*: injecting sufficient current for collective instabilities to produce microscale fluctuations, resulting in charged particles being radially transported across field lines [19–22].

In drift injection, the electric potentials that produce inward drifts over some segments of a particle’s trajectory correspondingly produce outward drifts during other sections [18]; for spatially and temporally localized pulses of charges (e.g., from a buffer gas trap [23]), however, the transient application of injection potentials is expected to produce inward fluxes that greatly exceed outward fluxes. Edge injection involves turbulence [22] that arises at collective densities; without these self-induced fluctuations to break the conservation of canonical momentum, particles do not penetrate radially on average over a toroidal rotation [24, 25]. Hence, both of these are intrinsically perturbative processes, but they have both nevertheless resulted in subsequent long trapping times [21, 26]. There is also the potential to combine these injection schemes, e.g., edge injection from a heated filament could be used to create a dense electron plasma, with a small Debye length, into which pulses of positrons could be drift injected. $\textbf{E}\times B$-drift injection of positrons into electron-generated space charge has been demonstrated both numerically [27] and experimentally [28].

Apart from the goal of producing a magnetically confined pair plasma [10, 13], injecting even a pulse of positrons much smaller than the electron population would extend the study of positronium-forming three-body processes (radiative and three-body recombination) from $< 0.005$ eV regimes [29] to temperatures of a few eV. Sky maps of 511-keV emission [30] suggest that recombination and subsequent annihilation in warm interstellar clouds could play a role in the galactic annihilation signature [31, 32]. Since magnetic confinement allows the positrons to keep passing through the electron cloud, appreciable signals could be obtained with achievable numbers of positrons and sufficiently long confinement times. (The magnetic fields required for confinement are, however, much higher than the nT interstellar field.)

A dipole magnetic field is a simple example of an omnigenous confinement geometry, and it is realizable with a minimal setup based around a supported permanent magnet. Such setups—sometimes called “terrella” (in reference to the Earth’s magnetic field)—have been fruitfully employed in a variety of fundamental plasma physics studies, with recent implementations still offering novel topics of study [33–36] even though the approach dates back to the turn of the 20th century (as reviewed by Rypdal and Brundtland [34]).

We report here on a series of experiments and simulations that use a permanent magnet-based dipole trap to advance our understanding and control of injection, confinement, and diagnosis of low-density collections of electrons and positrons. Drift injection and edge injection have been used to transport low-energy (eV-scale) electrons into the trap; experimental and numerical studies with positrons have been used to elucidate single-particle confinement times in the same trap; and finally, a gamma detector array has been commissioned, which in subsequent experiments will be used to measure the annihilation of a pulse of positrons injected into an electron cloud.

## Methods

### The supported dipole trap

**Fig. 1:**
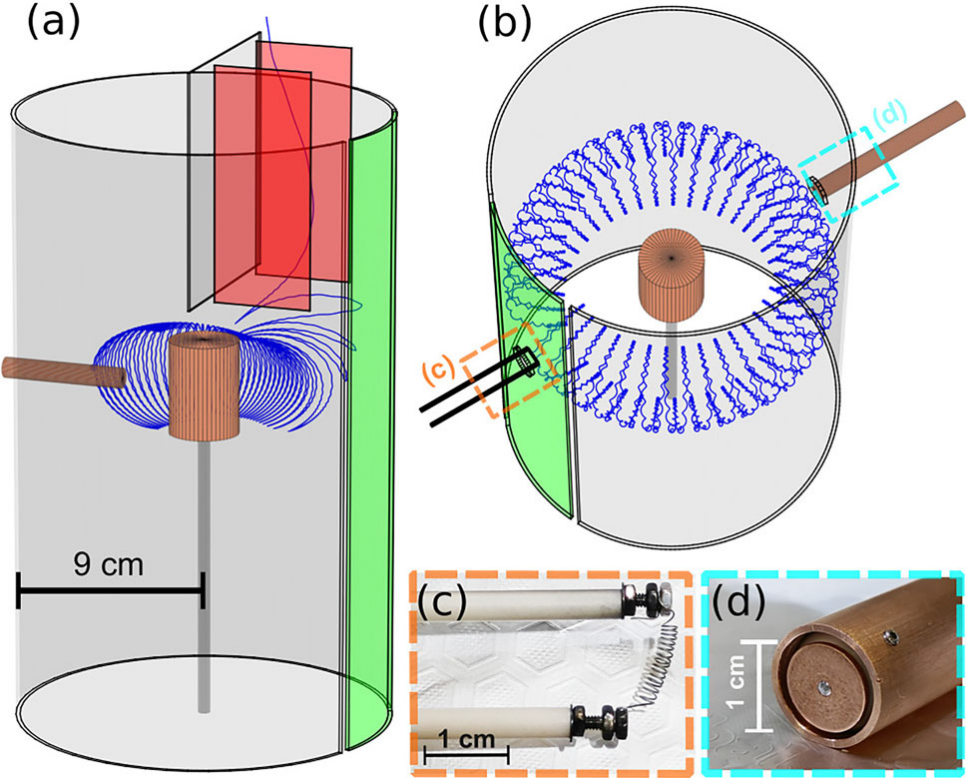
Setups for two different varieties of injection experiments in the supported dipole trap, with a single electron trajectory depicted for each (blue paths). **a** During drift injection, the $\textbf{E}\times B$ plates (red), the section of the wall between them (green), and the magnet case are biased. The primary diagnostic is an insertable target probe that intercepts the orbits of particles that, after being successfully injected, have toroidally drifted around to the opposite side of the trap. **b** During edge injection, a tungsten filament **c** emits electrons, the resulting space charge can be detected with a wall probe **d**, and the magnet case can be used for measuring currents

**Fig. 2:**
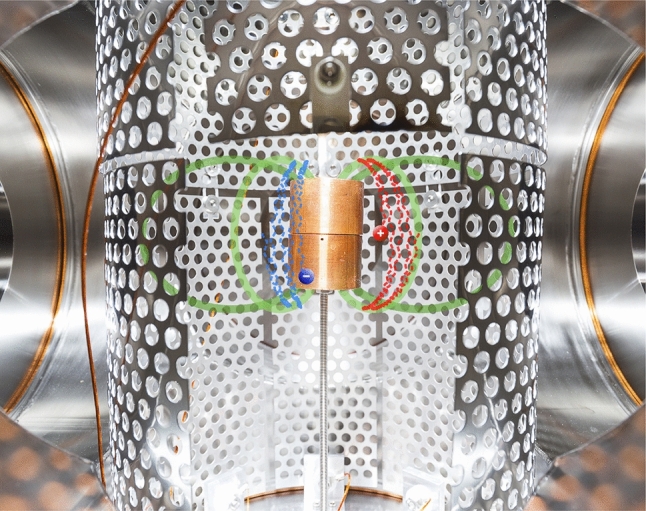
Graphic illustrating trapped charged particle motion in the magnetic field of the supported permanent magnet. Partial trajectories of a 5-eV electron and positron (blue and red dashed lines, ending in labeled dots), as well as selected magnetic field lines (green), were calculated from a model of the experiment geometry and then digitally merged with a photograph of the experiment, using the dimensions of the magnet case for scale

For this simple dipole trap (shown in Figs. 1–2), the confining magnetic field is supplied by a supported permanent magnet (0.5–0.6 T at its poles), installed in a six-way vacuum cross [14]. The magnet is enclosed in a close-fitting copper case (radius $r=1.65$ cm) to which a bias can be applied. The outer boundary of the “confinement region” (in which low-energy charged particles execute characteristic trapped orbits, as illustrated in Fig. 2 and described in more detail below) is limited by a cylindrical electrode wall at a radius of $r=9$ cm. (Different versions of the electrode wall—with different numbers and sizes of sections—can be installed to facilitate different desired perturbations.) On the horizontal midplane, the field strength ranges from $\sim$3 mT at the wall to $\sim$130 mT at the magnet case. Background pressures are typically in the $10^{-6}$–$10^{-5}$ Pa range.

For drift injection (Fig. 1a), two rectangular “$\textbf{E}\times B$ plates” are biased to opposite potentials (typically around 200 V each), creating an electric field perpendicular to the guide magnetic field along which charged particles approach the dipole from the beam line above. Upstream steering coils (not pictured) are used to adjust the position of the incoming beam within the beam line. The magnet and a vertical section of the wall are typically biased repulsively, above the beam energy, while the rest of the wall is grounded; the resulting three-dimensional electrostatic potential causes particles to transition to transiently trapped orbits, drifting toroidally around the trap. (Before they complete a full toroidal rotation period, particles will drift back out again [14, 18], unless the $\textbf{E}\times B$ plate and wall potentials are switched off in the meantime.) For edge injection (Fig. 1b), electrons are produced by thermionic emission from a thoriated tungsten filament with 15 windings and a wire diameter of 0.25 mm (Fig. 1c). The filament spans a 5-mm radial extent to increase the emitting surface toward the toroidal drift direction of the electrons.

Trapped charged particles complete three motions in a dipole field, as shown in Fig. 2: they gyrate around the magnetic field lines, they bounce back and forth along the poloidal magnetic field, and they drift toroidally. When the electric and magnetic fields vary gradually compared to the time and length scale of the motions, there are up to three associated adiabatic invariants. This includes the requirement that the Larmor radius $r_\textrm{L} = m v_\perp /(qB)$ be much smaller than the scale length of the magnetic field $L_\textrm{B}=B/\nabla B$. ($L_\textrm{B}=r/3$ along the midplane of an ideal point dipole, for example.) The effective potential energy experienced by the particles along the field is then described by $U = q\phi + \mu B$, with the magnetic moment $\mu =m v_\perp ^2/(2 B)$ corresponding to a particle’s gyromotion conserved (the “first” adiabatic invariant) [37]. $\phi$ is the electrostatic potential, *B* is the magnetic field magnitude, and *q*, *m*, $v_\perp$ ($v_\parallel$) are the particle charge, mass, and velocity perpendicular (parallel) to the magnetic field vector. When the magnet case is biased, trapped particles may be categorized as magnetically mirroring or electrostatically reflecting; the latter would be in the “loss cone”—i.e., have a small enough pitch angle $\theta = \text {arctan} (v_\perp /v_\parallel )$ to reach the magnet—if not for the magnet bias.

Adiabaticity-breaking frequently leads to transport and/or confinement losses. It may be caused, e.g., by high-frequency field fluctuations or collisions with background neutrals. The latter mechanism has been proposed as the primary loss mechanism in previous positron confinement experiments [26]. Elastic scattering leads to both velocity space diffusion (via which particles that were previously mirror trapped can scatter into the loss cone, when the magnet is not sufficiently repulsive) and spatial diffusion (with step sizes of order $r_\textrm{L}$). Spatial diffusion can transport particles toward the magnet (where the step size decreases, but the loss cone increases) or toward the wall. These processes occur at time scales much slower than the toroidal orbit.

### Diagnostics

Injected particles can be diagnosed with a multi-purpose probe (Fig. 1d) consisting of a 15-mm-diameter copper tube and a 1-cm-diameter copper disk, mounted to a PEEK cylinder inside the tube. A linear manipulator allows the probe to be radially translated through a hole in the outer electrode wall. When translated into the confinement volume, the copper rod serves as a target probe, blocking the charged particle orbits, and the current arriving on the copper rod is measured [28].

When fully retracted, the surface of the probe is positioned approximately flush with the end of the tube and tangential to the inner surface of the wall. Fluctuations in the charge distribution can be detected with a wall probe [38, 39]; the tube is electrically grounded, and the voltage signal on the copper disk produced by incident or induced charges is recorded. Fluctuations can also be measured by recording the voltage on the magnet case. These were acquired with a 200-MHz oscilloscope with 1 M$\Omega$ input (100 $\Omega$) impedance for the wall probe (magnet case).

The positron numbers injected into dipole fields to date are still well below the collective limit and do not produce fluctuations. However, the annihilation of positrons also presents diagnostic opportunity, potentially offering a sensitive “probe” of particle confinement times and spatial loss patterns. Confinement studies to date have typically involved only one or two gamma detectors (but were still able to determine trapping times via repeated experiment cycles [14, 26]). We now seek to better exploit this opportunity using an extended gamma-detector array [40] described in Sect. 5.

### Full-orbit trajectory simulations

Single-particle calculations are a powerful tool for interpreting and developing experiments, especially when working with low-density systems. For this work, we continue to utilize a set of simulation tools, developed in-house [41, 42], that combines an electrostatic potential solver and a full-orbit-resolving particle pusher (using a “leap frog” or Boris method [43]) with three-dimensional models of the electrodes and coils used to apply electric and magnetic fields in the experiment. The trajectories plotted in Figs. 1 and 2 are representative results for the respective initial conditions (i.e., starting positions and velocities).

For a collection of particles, initial spatial and velocity distributions in the simulation can be based on measured or nominal beam properties (spatial extents and energy spreads) when these are known, or they can be scanned as free parameters. Electrode switching—time-dependent evolution of the electric potentials—and a simple collision model can also be included. Simulations using these tools have been able to reproduce and predict key features of dipole and injection experiments, as well as to fill in “gaps” between diagnostically available quantities [18]. In this work, they inform interpretations of the experimental data (e.g., in Sects. 3.1 and 4.1), and they are the basis for a systematic study of different factors influencing the confinement time Sect. 4.2.

## Electron injection

### Drift injection

Over the last decade, $\textbf{E}\times B$ drift injection of positrons into a dipole field has been improved from 38% efficiency [14] to 100% [15], for a 5-eV beam with a temperature of $\sim 2$ eV and a spatial extent of $\sim 3$ mm [15, 44]. (For a wider beam, with a non-circular cross-section of 6–12 mm at FWHM, the efficiency peaked at 60–70% [17] [The technique has also been used with an electron beam produced by a hairpin tungsten filament positioned behind a Wehnelt electrode [16], albeit with low injection efficiency, due to wall and magnet electrodes being biased positively for the efficient co-injection of positrons.

**Fig. 3:**
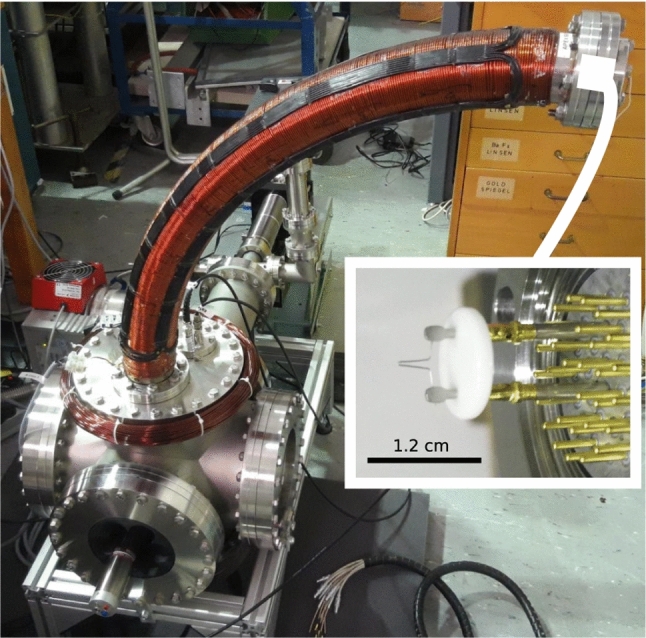
Photographs of the setup for electron drift injection show the hairpin filament (inset), which is installed on the flange upstream of the beam line curve (quarter circular vacuum pipe and solenoid), with its integrated steering coils (black wires), as well as the boost coil (wrapped around the flange below)

Recent experiments employed a bare hairpin tungsten filament, installed 3–4 cm upstream of a beam line curve (i.e., a $90^{\circ }$ bend with a 50-cm radius of curvature, guided by a 7-cm diameter solenoid), as shown in Fig. 3. The other end of the bend was installed $\sim$10 cm above the top of the $\textbf{E}\times B$ plates; there, a 25-cm-diameter “boost” coil supplied a supplemental guiding field from the bend into the region of the dipole trap. The filament was biased to 6 V relative to the grounded chamber, with an emission current of $\sim 2\,\upmu$A (stable after $\sim$5 min). Although the motion of the emitted 6-eV electrons is not adiabatic in the 1.0- to 1.5-mT magnetic field around the emitter ($L_\textrm{B}$ being comparable to $r_\textrm{L}$), electrons successfully injected into the bend subsequently experience reasonably adiabatic guiding, in *B* of 3–8 mT.

Two pairs of steering coils, extending over the length of the bend, adjust the transverse position of the electron beam in the beam line. The currents in these coils are denoted by the direction they move the beam in the dipole field coordinate system: toroidal $I_\theta$ and radial $I_\textrm{r}$. Injection biases were chosen to be comparable in magnitude (but opposite in polarity for the magnet and wall segment) to those used previously for lossless positron injection (e.g., [15]): magnet at $-8$ V, wall segment at $-20$ V, $\textbf{E}\times B$ plates at $\pm 217$ V.

**Fig. 4:**
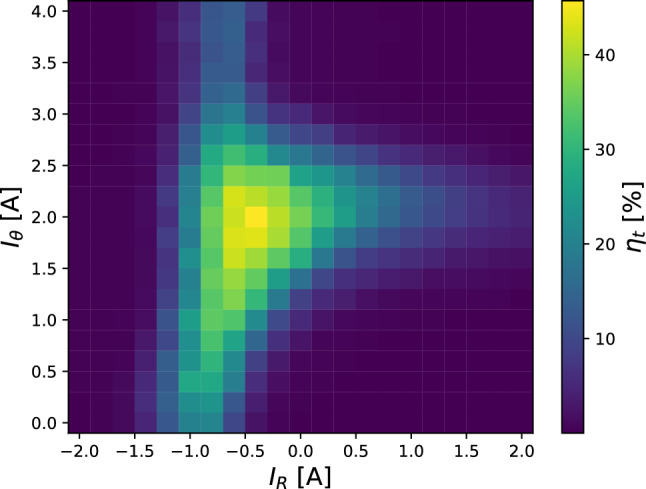
Efficiency $\eta _\textrm{t}$ of drift injection of electrons into the dipole field as a function of the two steering coil currents ($I_\textrm{r}$ and $I_\theta$), with other parameters (e.g. electrode biases) held constant

The resulting efficiency $\eta _\textrm{t} = I_\textrm{t}/I_\textrm{e}$ of the injection, defined as the ratio of the current arriving on the target probe $I_\textrm{t}$ to the emission current $I_\textrm{e}$, is plotted as a function of steering coil currents in Fig. 4. Compared to previous high-resolution measurements (e.g., fig. 8a,b of Ref. [28]), the contours of injection efficiency have similar triangular shapes with a peak of high efficiency in the center. However, the highest efficiency is only $\sim 45$%. A scan of the target probe position showed that the electrons are primarily at large radii, close to the wall. Additionally, another 35% to 40% of the emitted electrons intercepted the wall, rather than the target probe.

These features are likely attributable to the incoming electrons having a much broader distribution of pitch angles than beams used in previous work. Particles with larger pitch angles—i.e., larger $v_\perp$ and therefore larger $\mu$, for a given kinetic energy and magnetic field—are restricted by the three-dimensional effective potential to larger radii of the trap, where *B* and $\phi$ are lower, and they generally drift to the wall earlier in their toroidal transit [18]. Perpendicular energy spread is therefore a vital property of any incident particle beam that one aims to inject—at least when injection efficiency is important (typically the case for positrons, though not necessarily electrons) and/or when deeper radial profiles are desired. This is a further motivation for future experiments to employ a buffer gas trap system, where positrons will be accumulated and cooled upstream of the dipole trap [45, 46].

### Edge injection

Edge injection and subsequent confinement of electrons have previously been studied in a 0.01 T dipole field of a supported current ring [20] as well as a 0.5 T dipole field of a levitating current ring trap [22]. The electron source was a $\textrm{LaB}_6$ cathode, biased with respect to an anode gate at chamber ground. In both experiments, wall probes picked up a broad frequency range during injection. Once the emitter was turned off, the wall probe signal settled into a steady oscillation persisting for several hundred ms (supported) or seconds (levitated). The oscillation was non-sinusoidal with a spectral content made up of a dominant frequency and several harmonics. The dominant mode frequency scaled linearly with the inverse of the magnetic field $B^{-1}$ and the voltage bias of the internal conductor [20].

Our permanent magnet trap had previously been filled with appreciable electron space charge by means of a filament with a large radial extent of over 3.3 cm in the equatorial plane [28]. Electrons continued to interact with and be absorbed by the filament, preventing the observation of their collective modes during confinement. Here, we report on injection of electrons from the thermionic emission of the edge filament (Fig. 1c). The filament was heated with current $I_\textrm{h}$, resulting in emission current of a few mA. Results shown here used $I_\textrm{h}=4.8$ A; the behavior is similar over a range of $I_\textrm{h}=3.8$–5A. The inner filament edge was positioned at $r=6.85$ cm; at this radius  90% of the emission current is absorbed by the grounded magnet. The remaining electrons become mirror trapped and drift toroidally around the magnet. Both the magnet case and the wall probe pick up signals in the MHz frequency range during and after electron emission.

**Fig. 5:**
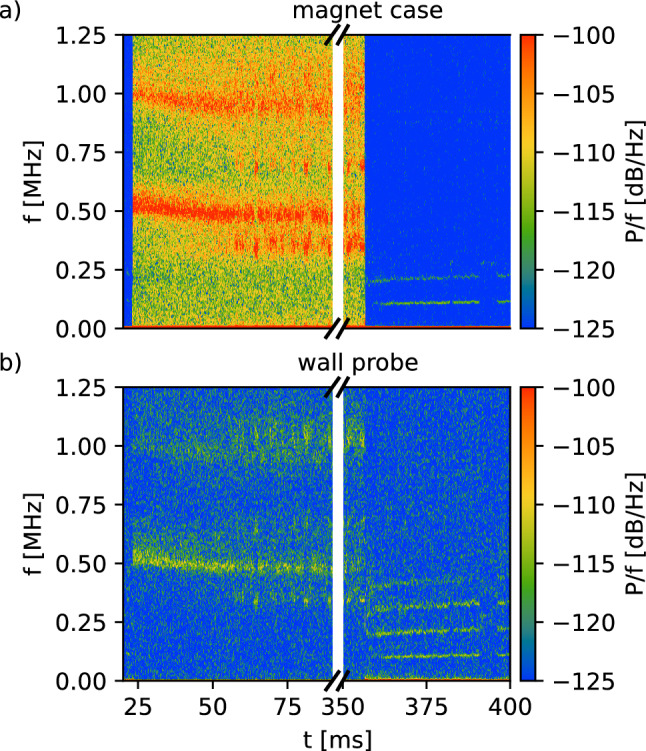
Power spectrum for the signals on the **a** the magnet case and **b** the wall probe during injection and confinement. The emitter bias is switched from ground to $V_\textrm{e}=-50$ V on at $t=23$ ms, initiating the injection phase. The emitter is grounded at $t=357$ ms, initiating the confinement phase. In order to show the dynamics around the bias switching, there is a jump in the time scale, marked with ‘//’

Each experiment cycle can be divided into the injection and the confinement phase. Figure 5 shows the power spectra of signals measured on the (a) magnet case and the wall probe (b) over a cycle. The injection cycle is initiated by switching the emitter bias $V_\textrm{e}$ from ground to a repulsive potential of up to 60 V ($V_\textrm{e}=-50$ V in Fig. 5) at $t=23$ ms. Broad power peaks form around 500 kHz and 1 MHz. The frequency of the peak declines as the dipole trap fills with electrons. After $\sim$25 ms of emission, the signals reach a quasi-steady state, with repeated transient disruptions of the peak signals. Despite the large difference in toroidal angle coverage of the symmetric magnetic case and discrete wall probe, the measured frequencies are nearly identical.

At 357 ms, the filament is grounded, interrupting the emission current and initiating the confinement phase. Higher frequencies disappear and several discrete frequencies <500 MHz appear, with a spectrum on both the wall probe and magnet case resembling the mode seen in previous dipole experiments, which was identified as a (toroidal) diocotron mode. It is therefore notable that this is seen on the toroidally symmetric magnet case as well as on the wall probe; this could suggest coupling between the toroidal mode and the poloidal motion of the electrons.

**Fig. 6:**
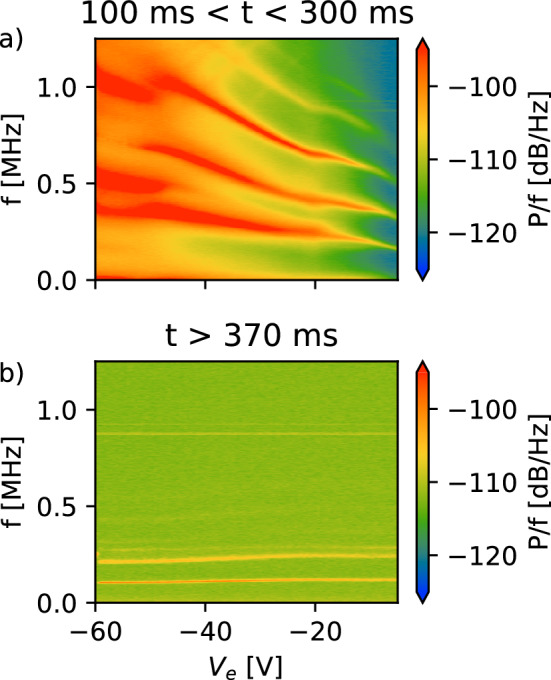
Emission bias $V_\textrm{e}$ dependence of the frequency spectrum picked up on the magnet case **a** while the emitter is biased (injection) and **b** after the emitter has been grounded (confinement). The current through the emitter is $I_\textrm{h}=4.8$ A

Figure 6 shows the frequency content of the magnet case signals as a function of the emitter bias $V_\textrm{e}$. During emission (panel a), the dominant frequencies depend on emitter bias; discontinuous jumps in the dependence at larger bias magnitudes ($> 40$ V) were reproducible over repeated emission bias scans. During the “confinement” phase (panel b), after the emitter is grounded, there remains only weak dependence on what the emitter bias had been in the injection phase, with the dominant frequency varying by less than 20 kHz over the entire range.

These features are not yet fully understood and will be among the topics of further studies into the generation and evolution of pure electron plasmas in dipole fields. Experiments will be conducted not only in this trap but also in a compact levitated dipole trap based on a high-temperature superconducting coil [10], which has just gone into operation; theoretical and numerical modeling [47] will help guide and interpret the experiments.

## Positron confinement

### Experimental results

The permanent magnet trap was built not to maximize confinement times but for use in prototyping techniques for injection and diagnostics, so as to help inform the design process for subsequent, higher-field traps for pair plasma studies [10]. It is nevertheless worthwhile to develop an understanding of what loss mechanisms are dominant in this setup under various conditions, including for collections of charges in the single-particle regime, as is the case for the positron experiments to date.

When the $\textbf{E}\times B$ plates are left biased continuously, drift-injected charged particles are only transiently confined for less than a single toroidal orbit of the trap, ${\mathcal {O}}(10\,\upmu \text {s})$; simply switching off $V_{E\times B}$ was seen to extend the longest characteristic time scale $\tau$ on which positrons could be observed “leaking out of the trap” (i.e., elevated annihilation gamma count rates, measured as a function of the time elapsed since the start of a confinement period) by two orders of magnitude, to ${\mathcal {O}}$(ms) [14]. Those initial experiments were conducted with the magnet case negatively biased, though, which has the side effect of “opening the loss cone” (increasing the range of pitch angles that can reach the magnet). When the magnet case was grounded or positively biased, $\tau$ increased another order of magnitude, to ${\mathcal {O}}$(10 ms) [17, 48].

Additionally, it was discovered that positrons remained in the trap even longer than could be unambiguously seen with that original measurement scheme. The small solid angle coverage (of a single gamma detector located 20-30 cm from the magnet) meant that the “leakage” signal could not be detected above the background if positrons were better confined; deliberately “dumping” the trap, however—by switching the $\textbf{E}\times B$ plates back on after some “hold” time—resulted in a clear gamma burst from the annihilation of those long-trapped positrons. Operating in “fill–hold–dump” cycles, in which the confinement time of nominally $\sim$6-eV positrons was calculated from the dependence of “dump” counts on the hold duration, yielded $\tau$ of $\sim$200 ms for a grounded magnet, $\sim$400 ms for a 5-V magnet bias, and $\sim$1.5 s with an 8-V magnet bias [17]. This last case corresponds to hundreds of thousands of toroidal orbits.

**Fig. 7:**
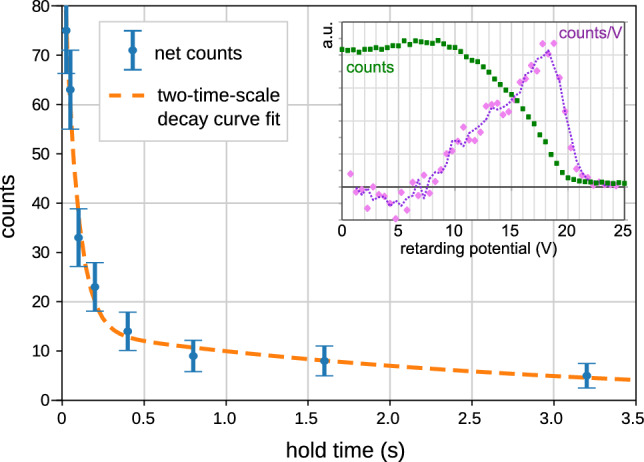
Confinement measurements of low-energy positrons around the supported permanent magnet indicate at least two well-separated timescales for loss mechanisms. Inset: A retarding potential was used to measure the positron beam’s initial parallel energy distribution (“counts” curve); these data and its numerical derivative (“counts/V” curve) are both plotted in arbitrary units

Here, we report on further investigations of extended confinement, this time for a positron beam with a higher and broader parallel energy distribution (shown in the inset of Fig 7) than previously used. As in previous experiments, the positrons were supplied by the NEutron-induced POsitron source MUniCh [49], operated by FRM II at the Heinz Maier-Leibnitz Zentrum (MLZ), Garching, Germany. (These measurements were made prior to the ongoing, multi-year break in operation.) The spatial distribution of the drift-injected beam, as measured with the insertable target probe, was located between radii of 5.5 and 7 cm. The background pressure in the experiment was $3\times 10^{-6}$ Pa (mostly water) For the hold phase, the magnet bias was kept at 20 V (its setting for injection), and the entirety of the wall was grounded. The resulting numbers of “dump” counts are plotted, as a function of hold time, in Fig. 7; at least two distinct time scales are evident, one $\le 100$ ms and another on the order of seconds. (The curve fitted to the data is a weighted sum of two exponentials with components 0.078(±0.022) s and 2.8(±1.7) s, albeit underdetermined due to the overall low number of counts.)

The relatively large proportion of faster losses could be due to scattering into the loss cone, at least in part. Although the 20-V magnet bias was above the initial beam energy, the fast (sub-$\upmu$s) switching off of the $\textbf{E}\times B$ plates has been found to accelerate positrons in the vicinity to significantly higher energies [18]. Since, however, much of the positron population is above relevant energy thresholds for inelastic collisions (first electronic excitation energy, ionization potential, and threshold for positronium formation, as compiled in the supplementary material of [26]), there may also be more complex mechanisms at play. Relatedly, the longer (second-scale) component is a notable feature, given that higher-energy particles have larger step sizes for spatial diffusion.

Future experiments with different positron beam energies—preferably with low energy spreads and injected with a narrow radial spread and minimal perturbation from the switching, as could be achieved with pulses from a buffer gas trap [45, 46]—will seek to distinguish between various possible particle and energy loss processes and combinations thereof. These are best pursued with significantly better diagnostic capabilities (the development of which is detailed in Sect. 5) and significantly more trapped positrons. The single-detector measurement setup that is used for Fig. 7 required 4096 fill–hold–dump cycles per hold time, adding up to  11 h of measurement in total, and this yielded only $\sim$230 net counts. That inefficiency, combined with the scarcity of positrons (which has been especially acute lately), also points to simulations as an important avenue for understanding the mechanisms and time scales of confinement.

### Strategies for extending $\tau$

**Fig. 8:**
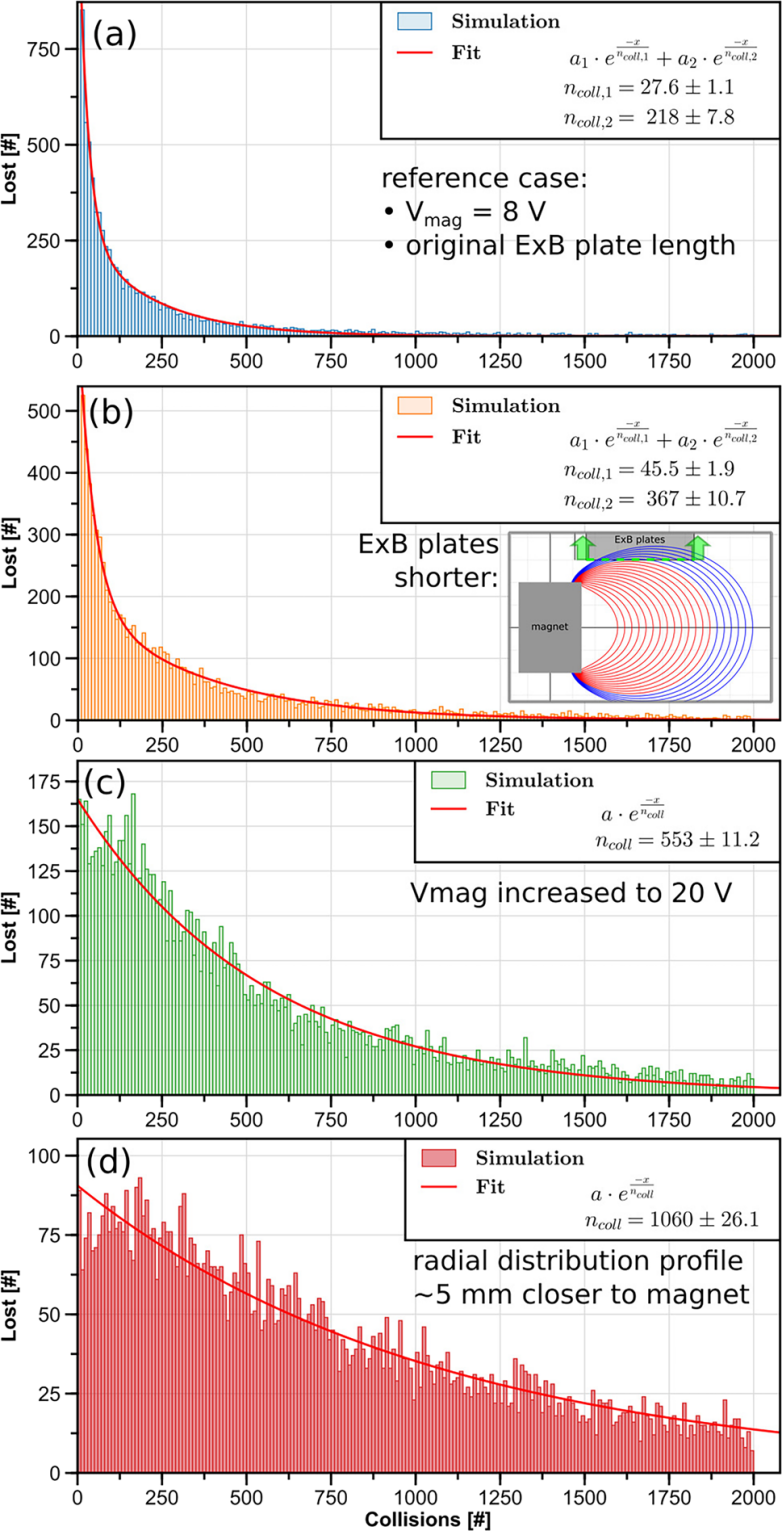
Histograms of 5-eV positrons lost as a function of the number of elastic collisions they have undergone, for four different confinement simulations

In single-particle simulations to investigate strategies for extending the confinement time, we used a 5-eV positron beam and assumed the main loss mechanism to be elastic scattering with residual gas. Some simplifications were made for computationally feasibility. First, elastic scattering was implemented by randomizing the direction of the positrons’ velocity vector. Second, it was necessary to conduct the simulation with an unrealistically high collision frequency (on order of 1 MHz) to reduce the simulation time to a reasonable duration. (The small time step needed to resolve the gyromotion makes it costly to simulate particles for more than 10 ms—the same order as the expected mean collision time in the experiment.) This artificially high collision frequency was still lower than the bounce frequency but higher than the toroidal rotation frequency; to correct for resulting missed encounters with the $\textbf{E}\times B$ plates or the shield plate, a positron was registered as lost if it reached the necessary height (regardless of toroidal angle).

Figure 8a shows the resulting histogram of positron losses versus numbers of collisions, for the reference case based most closely on experiments. The data is fit well by a two-component exponential decay function, where the fast decay (with a characteristic timescale of $\sim$28 collision periods) corresponds to particles scattering into the magnet loss cone, and the slow decay ($\sim$220 collision periods) corresponds to positrons spatially diffusing out to larger radii ($r>$7.1 cm), where they are likely to encounter the bottoms of the $\textbf{E}\times B$ plates.

Since longer confinement times are mainly limited by the plates, the next simulation uses plates that are 2 cm shorter (after verifying that this was not detrimental to the injection process). The results are shown in Fig. 8b. By merely shortening the plates, the confinement time could be extended by more than 65%. There are still two loss processes present: the faster ($\sim$46 collisions) is still the scattering of positrons into the loss cone of the magnet; the slower ($\sim$370 collisions) is now the outwards diffusion and the subsequent annihilation on the wall.

The third simulation case demonstrates full “plugging” of the loss cone by applying a higher potential to the magnet case, with results for a 20-V bias (Fig. 8c). The short-time-scale loss is eliminated, and the overall confinement time ($\sim$550 collisions) is increased 50% compared to the shorter plates alone, or by a factor of 2.5 compared to the reference simulation. Since changing the potential applied to magnet case would affect the radial distribution of the injected positrons, special care was taken to maintain the same radial distribution as before (as shown in 9) by adjusting the $I_\textrm{r}$ steering coil current adjustment.

**Fig. 9:**
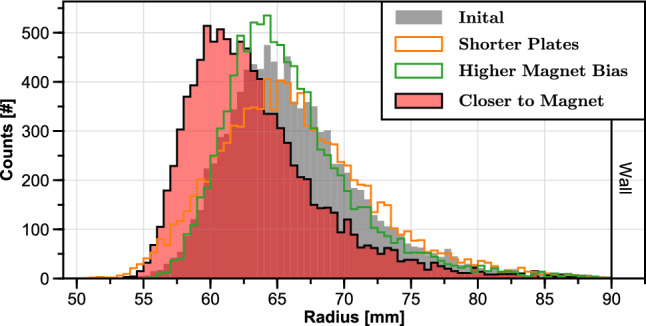
Radial spatial distributions of the positrons, for the four confinement results presented in Fig. 8

After shortening the plates and increasing the magnet case bias, the only loss channel remaining in the simulation was the outward diffusion of positrons, leading to loss on the wall. To further increase the confinement time, free parameters that affect the radial distribution were adjusted to inject positrons closer to the magnet. In Fig. 9, the peak shaded red illustrates how much the radial distribution is shifted inward when the magnet case bias is lowered to 15 V (still enough to repel positrons) and $I_\textrm{r}$ is set to −7 A instead of $-$6.2 A. This radial shift of just 5 mm significantly improved the confinement, as seen in Fig. 8d), to $\sim$1060 collisions—almost double the previous case.

The simulations thus point to strategies for significantly lengthening confinement times. In the future, we plan to combine these with experimental changes that will reduce the collision frequency (via better vacuum conditions) and the radial diffusion step size (by using lower-energy particles and/or stronger confining magnetic fields to reduce $r_\textrm{L}$).

## Annihilation-based diagnostics

Detection of annihilation gamma rays has been an indispensable tool for positron experiments in the supported dipole trap, despite the solid angle coverage having generally been quite low. Injection settings have been optimized using a scintillation detector with a field of view narrowly collimated onto the insertable target probe (after the net count rate was found to track the target probe current measurements) [15]. Confinement times were measured using a single detector viewing the entirety of the trapping region’s limiting surfaces [14]; counts had to be averaged over thousands of cycles to achieve usable signal-to-noise ratios.

Here, we present a significant upgrade to gamma diagnostic capabilities: an array of 25 uncollimated, 2.54-cm-diameter, bismuth germanate (BGO) detectors. With it, we demonstrate two different techniques that take advantage of the spatial correlations of positron annihilation gamma rays originating from a localized source:

1. *single-photon counting*: Since annihilation is an isotropic source, the number of counts at each detector $C_\textrm{i}$ will be approximately proportional to the inverse square of the distance from the source.
2. *coincidence*: Momentum conservation results in back-to-back 2$\gamma$ emission occurring approximately along a line. Coincident detection on two detectors indicates that the source is likely located on the line of response (LOR) connecting them.

In future experiments, a localized source may result from “dumping” electrostatically trapped positrons onto the magnet by grounding it (thereby giving a measure of the loss cone population) or from the annihilation of positrons that have radially diffused out to a limiter (e.g., a target slightly protruding into the trap from the electrode wall). We can use both single-photon and coincident LOR counting to locate such sources. The advantage of single-photon counting is that sources can be located without collimation, maximizing solid angle coverage and detected photons [50–52]. Coincident LOR counting, on the other hand, can locate sources with a few overlapping LOR due to the self-collimation to the LOR [53–55]; however, this self-collimation also limits the solid angle coverage.

**Fig. 10:**
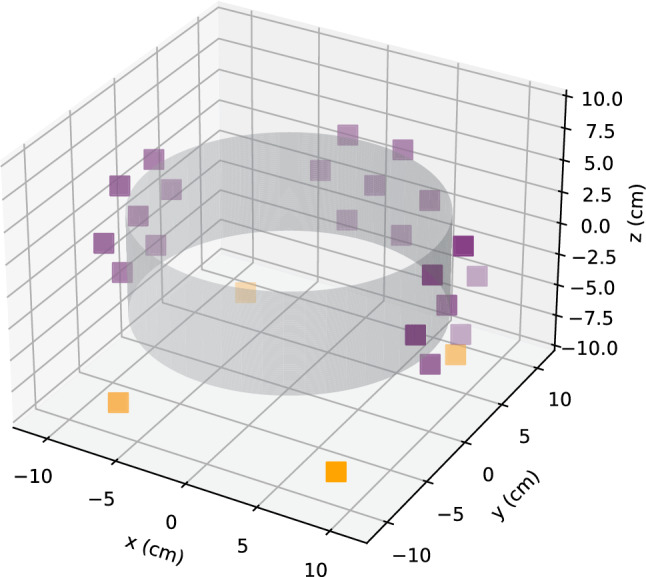
Positions of 25 detectors in re-entrant ports (purple) and in the corners below the chamber (orange). The magnet (not shown) is centered at the origin. The electrode wall (gray) is at $r=9$ cm

To demonstrate these techniques, we install our BGO-detector array around the chamber (Fig. 10) and use it locate a $^{22}$Na source offset from the magnet. Groups of seven detectors are placed inside three re-entrant ports; four detectors are arranged in a square below the chamber.

**Fig. 11:**
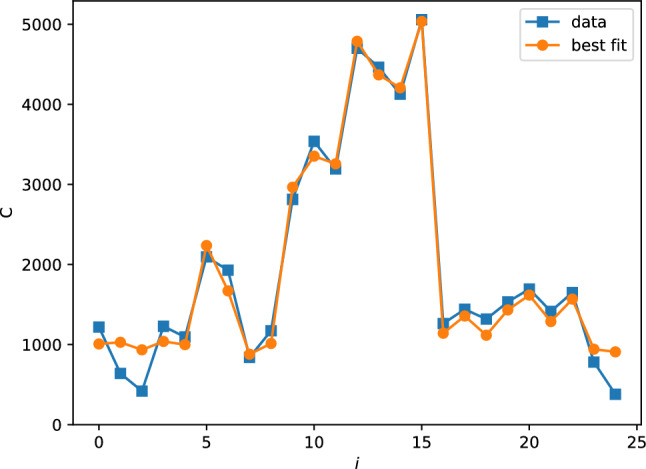
Distribution of $10^5$ single photon counts $C_\textrm{i}$ among the 25 detectors (blue rectangles), along with the best fit values (orange circles) for a point source

The expected number of single-photon counts $C_\textrm{i}$ can be modeled with the solid angle relationship for a point source along the axis of a cylindrical detector [56]:

1 $$\begin{aligned} C_\textrm{i} = A \left( 1 - \frac{\ell _\textrm{i}}{\sqrt{\ell _\textrm{i}^2 + \alpha ^2}} \right) , \end{aligned}$$

where $\ell _\textrm{i}$ is the distance of detector *i* from the source, $\alpha$ is the detector radius, and *A* is a constant related to the activity of the source. Fitting equation (1) to the distribution of counts across detectors can be used to determine the location of the point source. Figure 11 shows the distribution of $10^5$ single photon detections, as well as the number expected from the best fit for the source position, which was calculated to be $x=-4.7 \pm 0.2$ cm, $y=-1.3 \pm 0.3$ cm, $z=-1.9 \pm 0.3$.

**Fig. 12:**
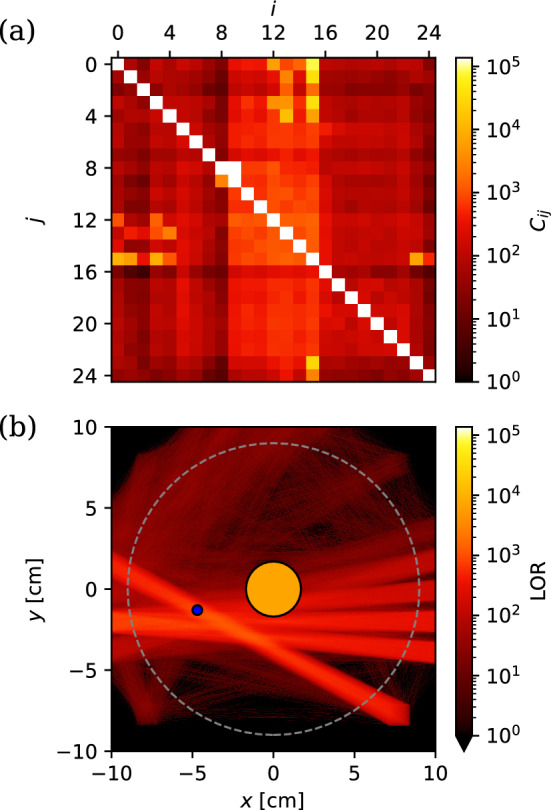
Coincident detections. **a** Distribution of $10^5$ coincident events across 600 detector pairings *ij* from a $^{22}$Na source placed offset from the magnet. **b** Lines of response between detector pairs, with brightness corresponding to the coincident events. The endpoints of the LORs are purposefully blurred to represent the finite detector size. The source position fitted from single-photon counts is marked with a blue circle. The electrode wall at $r=9$ cm is shown with a dashed gray line, and the magnet case is depicted by the orange circle

Coincident detection is possible with precise time stamping. The detector signals are digitized and processed with a field-programmable gate array to produce a list of all events, each with an energy and a time stamp. The time resolution of the system is 24 ns [40]. Figure 12 a) shows the distribution of coincident detections within 24 ns. More than 60% of coincident detections are on 5 of the 300 detector pairs with distinct LOR ($\text {LOR}_{ij} = \text {LOR}_{ji}$), indicating spatial localization. Plotting the lines of response (Fig. 12b) reveals a high density of intersecting LOR in the area around $-4$ cm$<x<-2$ cm, $-3$ cm $<y< -1$ cm, $-3$ cm $<z<-1$ cm. This is close to the single-photon-counting fit, with the small deviation attributable to the limited LOR resolution.

This newly commissioned detector array will enable much more efficient and sophisticated positron confinement studies than had been possible before, thanks to the greatly increased solid angle coverage, time resolution, and spatial localization of gamma emission. We anticipate gaining new insights into the rate of volumetric annihilation versus transport-driven losses, as well as into the evolution of the positrons’ distribution function (as manifested, e.g., by the loss cone content, which can be dumped onto the magnet).

As discussed earlier (Sect. 1), the annihilation of low-energy positrons embedded in a electron plasma is relevant to astrophysical sources of 511– keV gamma-rays. The predicted radiative recombination rate for a positron in a 1-eV electron plasma with densities of $10^{11}$–$10^{13}$ $\mathrm {m^3}$ is in the range of $\Gamma = 10^{-7}$–$10^{-5}$ $\mathrm {s^{-1}}$ [40]. The resulting number of coincident counts seen by detector pair *ij* is $C_{ij}\sim (1/3) \Gamma N_{\textrm{e}^{+}} \Omega _{ij} \eta _{pp}^2$, where 1/3 is the fraction of positronium annihilating to 2-$\gamma$, $N_{\textrm{e}^{+}}$ is the number of positrons injected into the electron plasma, $\Omega _{ij}$ is the total coincident solid angle, and $\eta _{pp}$ is the detection efficiency for gammas in the 511-keV photopeak. We would like to achieve a $C_{ij} \sim 0.1$ (i.e., about 100 volumetric annihilation events detected in 1000 cycles). For our setup—i.e., detector geometry as given above, expected orbit volume based on the trap geometry, photo-peak efficiency $\eta _{pp} \sim 0.4$ (from calibration measurements)—this would require $N_{\textrm{e}^+}$ of $10^7$ to $10^{9}$ positrons. The lower end of this is within the range of the buffer gas trap system (BGTS) planned for installation at NEPOMUC [46], while the upper end represents the content of the entire quasi-neutral pair plasma, for which additional positron accumulation stages are being developed [10, 57].

## Summary and outlook

We have reported on recent work in several areas that together advances our control and understanding of injection, confinement, and diagnosis of low-density collections of electrons and positrons in a simple magnetic dipole trap. Drift injection and edge injection have been used to transport low-energy (eV-scale) electrons into the trap, experimental and numerical studies have been used to elucidate single-particle confinement times in the same trap, and finally, a gamma detector array has been commissioned. In future experiments, we plan to combine these to study the annihilation of a pulse of positrons injected into an electron cloud.

## Data Availability

Data and software are archived on backed-up servers of the Max Planck Institute for Plasma Physics and/or Max Planck Computing and Data Facility and are available upon reasonable request.
